# A qualitative examination of the evolving role of sports technology in collegiate coaching

**DOI:** 10.3389/fspor.2025.1644099

**Published:** 2025-09-01

**Authors:** Mollie Brewer, Kevin Childs, Celeste Wilkins, Zachary R. Smith, Spencer Thomas, Kristy Elizabeth Boyer, Jennifer A. Nichols, Garrett F. Beatty, Daniel P. Ferris

**Affiliations:** ^1^ Department of Computer & Information Science & Engineering, University of Florida, Gainesville, FL, United States; ^2^ J. Crayton Pruitt Family Department of Biomedical Engineering, University of Florida, Gainesville, FL, United States; ^3^ University Athletic Association, Gainesville, FL, United States; ^4^ Department of Applied Physiology & Kinesiology, University of Florida, Gainesville, FL, United States

**Keywords:** sports coaching, sports technology, collegiate athletics, student-athletes, coach-athlete relationship, technology adoption, performance monitoring, human-centered computing

## Abstract

Coaches play a central role in shaping athlete performance and development. In collegiate sports, coaches must balance competitive goals with the broader needs of student-athletes. As technology becomes more available in sports, it is becoming increasingly embedded in the workflows and decision-making processes of coaching staff. While many recognize the growing presence of these tools in sports, there is limited understanding about how coaching staff select and integrate these tools into their professional practice. This study addresses this gap by investigating (1) the types of technologies that collegiate coaching staff use; (2) how coaches integrate those technologies into key coaching domains such as baseline testing, practice planning, and injury management; and (3) what motivates or hinders technology adoption in this environment. We conducted five semi-structured focus groups with 17 coaching staff members from National Collegiate Athletic Association (NCAA) Division I sports teams in the United States, representing men’s American football, men’s basketball, women’s basketball, women’s soccer, and women’s volleyball. Participants included coaches, athletic trainers, strength and conditioning staff, dietitians, sports scientists, and administrative staff. We provide an inventory of technologies in active use to support key aspects of coaching. Our findings show that when aligned with coaching goals, technology offers valuable support for decision-making, individualized student-athlete management, and coach-athlete communication. These findings also point to the importance of supporting coaching staff in managing the growing demands of technology use. By highlighting how collegiate coaching staff apply technology, this study deepens understanding of what technology integration in coaching looks like in real-world practice. The insights may offer valuable direction for scholars, coaches, and organizations who aim to strengthen coaching practice and athlete outcomes through thoughtful integration of technology.

## 1 Introduction

Coaches are among the most influential figures in an athlete’s career, shaping not only their performance but also their overall development and future opportunities (1). Effective coaching can enhance athletes’ confidence and long-term progress, whereas poor coaching can undermine abilities, erode trust, and increase the risk of injury (2–4). The coach-athlete relationship is therefore central to individual athlete development and broader team success (1, 5). This influence becomes even more important in today’s data-rich sports environment, where coaching staff are not only tasked with guiding athletes but are also central to translating data into informed decisions, such as linking force production to injury risk or synthesizing workload and recovery metrics to guide training plans (6).

Coaching has long been grounded in experience, intuition, and deep knowledge of the sport (7). Coaching involves teaching and refining athletes’ technical and tactical skills, structuring training, managing their workload and well-being, preparing for competition, and making real-time adjustments during play (4). Elite coaches have often led these practices through on-the-ground experience, refining new approaches before they are formally studied or supported by empirical evidence (8). Today, that experiential foundation is evolving alongside the growing presence of sports technology, which refers to tools or systems designed to collect, process, and deliver data related to athlete performance, health, or well-being, and applied within the specific context of sports (9). For simplicity, we sometimes refer to sports technology as “technology” throughout this article. Technologies such as GPS-based trackers, wearable sensors, and advanced video analysis platforms have introduced new ways for coaches to observe and assess their athletes (10). More advanced systems, including those using artificial intelligence, are increasingly positioned as sources of deeper insights for coaches through predictive modeling and pattern recognition (11, 12). As these technologies become more embedded in coaching workflows, there is a growing need to understand not just what technologies are available, but also how coaching staff integrate them within the context of daily coaching practice.

Recent studies have begun to explore how coaching staff perceive and adopt sports technologies, particularly within elite or professional settings. One of the earliest studies in this area, conducted by Liebermann et al. (13), found that while many experienced coaches generally recognized the value of technology and felt comfortable using it in other contexts, fewer than two-thirds of them reported using it as part of their athlete training process. This gap between recognizing potential and applying technology in practice remains evident in more recent work. In a scoping review of athlete monitoring literature, Timmerman et al. (14) synthesized studies, showing that although coaching staff view technologies to support athlete monitoring as valuable, their use was often constrained by data complexity, time demands, and limited resources. Similarly, Neupert et al. (15) found that monitoring practices often depended on coach buy-in and alignment among staff, noting that while most coaching staff believed monitoring technologies could enhance athlete performance, many had worked in successful high-performance sporting environments that did not utilize them. Together, these studies highlight the importance of examining not only how coaching staff use technologies, but also what motivates or hinders adoption in practice. We extend this work by exploring how coaching staff in collegiate sports integrate technologies across multiple domains of coaching–not limited to athlete monitoring–and the factors that influence their use, including the motivations that drive adoption in situ and the barriers that shape whether technologies are meaningfully applied.

While prior work has largely focused on elite or professional sports, the collegiate sports setting presents a distinct and understudied context. In the United States, universities invest heavily in state-of-the-art athletic facilities (16), sports performance technologies (17), and athlete recruitment efforts (18), creating immense pressure for coaching staff to deliver results. Moreover, the coach-athlete relationship is unique in this setting because collegiate student-athletes occupy a position between youth and professional sports. Unlike in youth sports, where parents play a mediating role, or in many professional sports, where athletes operate with greater autonomy, collegiate student-athletes are deeply dependent on their coaches for athletic development and academic support. In this high-performance environment, decisions about technology adoption and data use are tightly coupled with athlete development and future opportunities. Despite the growing role of technology in this context, little is known about how collegiate coaching staff engage with sports technologies in practice. To address this gap, we ask the following research questions (RQ):

• RQ1: What types of technologies are currently being used by coaching staff in elite collegiate sports?
• RQ2: How are technologies being used by coaching staff in key coaching domains, such as baseline testing, practice planning, competition preparation, and injury recovery?
• RQ3: What goals and motivations drive the adoption of these technologies, and what barriers limit their effective use?

To explore these questions, we conducted five semi-structured focus groups, each representing staff from a National Collegiate Athletic Association (NCAA) sports team. Across the five focus groups, 17 total coaching staff members shared their experiences with and perspectives on integrating sports technologies into their day-to-day coaching practices. This study contributes a practice-centered understanding of how collegiate coaches integrate technology into their coaching methods, with a focus on both the benefits and barriers to adoption. By surfacing the value and limitations of technology, this article offers an inside look at how elite collegiate coaches leverage their expertise, navigate organizational structures, and manage relational dynamics to make technology meaningful in practice. These insights can inform researchers, coaches, and athletic organizations aiming to implement technology more effectively and in ways that align with the realities of coaching practices.

## 2 Materials and methods

### 2.1 Study design and participants

To answer our research questions, we conducted five semi-structured focus group interviews with 17 staff members from a single NCAA Division I university in the southeastern United States. We recruited participants through existing relationships with the university’s athletic department. An athlete department staff member assisted in coordinating participation by contacting relevant coaching staff and scheduling the focus group meetings. Our participants represented five collegiate sports teams: men’s American football, men’s basketball, women’s basketball, women’s soccer, and women’s volleyball. Each focus group included staff who primarily worked with the same sport team. Participants included coaches, athletic trainers, strength and conditioning staff, dietitians, sports scientists, and administrative staff. One dietitian participated in both Focus Group 1 and Focus Group 5, reflecting the reality that some staff serve multiple teams within the athletic department. Table 1 summarizes the composition of each focus group, including gender and role distribution.

**Table 1:** Summary of focus groups and participants.

Focus group	Number of participants	Men	Women	Roles represented
1	3	3	0	Dietitian${}^{a}$, Athletic Trainer, Strength & Conditioning Coach
2	6	6	0	Head Coach, Assistant Coach, Athletic Trainer, Strength & Conditioning Coach, Sports Scientist, Administrative Staff
3	3	1	2	Assistant Coach, Dietitian, Strength & Conditioning Coach
4	3	1	2	Head Coach, Athletic Trainer, Strength & Conditioning Coach
5	3	3	0	Dietitian${}^{a}$, Athletic Trainer, Strength & Conditioning Coach

Specific sports teams (e.g., women’s soccer, men’s basketball) are not listed to avoid potential identification of participants.

${}^{a}$The same dietitian participated in both Focus Group 1 and Focus Group 5.

### 2.2 Data collection

We held each focus group in a private on-campus setting and recorded audio with participant consent. Each focus group included three to six participants and lasted approximately 30 min. The smaller size of some groups reflects NCAA coaching limits, which caps the number of countable coaches per team (e.g., four in soccer) (19). While teams may include additional staff, such as athletic trainers and strength and conditioning coaches, total coaching staff sizes remain relatively small. We began with roundtable introductions in which participants described roles and their primary coaching goals. This helped establish rapport and provided shared context for the group discussion. We then used a flexible set of guiding questions developed by the authors, drawing on our interdisciplinary expertise in human-centered computing, sports science, and applied experience in athletics. The questions explored participants’ use of technology in coaching practices, interpretation of metrics, internal collaboration, communication of insights with student-athletes, challenges, and desired improvements. The semi-structured format allowed flexibility, enabling follow-up questions to explore certain topics in greater depth as needed. We transcribed all audio recordings verbatim for analysis, resulting in 79 pages of transcripts. The university’s human subjects Institutional Review Board and the university’s athletic research review board approved the study (Protocol #: ET00023249).

### 2.3 Data analysis

We used a two-stage qualitative analysis to address our research questions. To address RQ1, we performed a content analysis (20) to systematically identify and categorize each type of technology mentioned within each focus group. We reviewed all transcripts to extract references to specific technology, primarily identifying commercial product names mentioned by participants (e.g., Catapult, ForceDeck, InBody). We then grouped these technologies into categories based on their primary function (e.g., GPS/IMU-based tracking, muscular and force assessment). For each category we summarized the purpose and use case in coaching based on how participants described the technology. We also noted the frequency with which each category appeared across focus groups to identify the most commonly discussed technologies. This process allowed us to capture the breadth of technology use before engaging in a deeper thematic exploration of their application in coaching contexts.

To address RQ2 and RQ3, we used thematic analysis following the six-phase framework provided by Braun and Clarke (21). This article presents a secondary analysis of an existing dataset (6) with a new focus on how technology in collegiate sports supports specific coaching domains and what shapes its use in practice.

We began by reviewing the transcripts in full to develop familiarity with the data (*Phase 1: familiarization*). A new subset of authors revisited the prior codebook from the primary analysis, reorganized and extended the codes to align with the research questions, and added new codes when necessary (*Phase 2: coding*). We held iterative team discussions to group and refine codes into themes related to practical coaching domains (e.g., establishing baselines and benchmarks, practice planning, injury recovery) and factors influencing the adoption of technology (e.g., perceived value, staffing needs, athlete engagement) (*Phases 3–5: developing, reviewing, defining, and naming*). We finalized the themes collaboratively and organized them into a narrative, integrating both content and thematic analysis to report our findings (*Phase 6: writing up*).

## 3 Results

This section presents the findings from our analysis. Section 3.1 outlines the types of technologies used by collegiate coaching staff (RQ1). Section 3.2 examines how these technologies are integrated into key coaching domains (RQ2). Section 3.3 explores the factors that drive the adoption of these technologies, as well as the barriers that limit their effective use (RQ3). Throughout the results section, (n) refers to the number of focus groups that discuss a given theme, not the number of individual participants.

### 3.1 Technology inventory in collegiate athletics

Collegiate coaching staff utilize a range of technologies to support performance optimization and athlete management. Across the five focus groups, participants identified 28 different technologies. The extent and type of technology usage varied among coaching staff and across focus groups. One focus group reported using as many as 23 technologies, while another reported as few as 6. While certain types of technologies were consistently mentioned across all groups, others were referenced only in specific contexts or by particular focus groups. Factors influencing these patterns are discussed further in Section 3.3.

From our findings, seven categories of technologies emerged, reflecting the range of tools currently integrated into collegiate coaching practices (see Table 2). Among these, three main categories of technology were most commonly mentioned across all five focus groups. They are technologies for tracking movement and assessing external workload [e.g., Global Positioning System (GPS) and Inertial Measurement Unit (IMU)], tools for assessing strength and neuromuscular function (e.g., force plates), and platforms for capturing biomechanical and motion-related data. Coaching staff described using these technologies to collect and process data that generated metrics such as player load (a composite metric that captures the intensity and duration of physical activity, often calculated from movement data such as accelerations, decelerations, and distance covered), sprint velocity, and indicators of neuromuscular fatigue or injury risk.

**Table 2:** Technology inventory in collegiate athletics.

Category	Purpose	Example technologies	Use case in coaching
GPS/IMU-based tracking	Quantify external load and movement characteristics such as distance, speed, and accelerations	Catapult (Melbourne, Australia)	Used to assess total workload, manage player fatigue, and adjust practice intensity.
Muscular & force assessment	Measure jump mechanics, strength imbalances, and neuromuscular performance	ForceDecks, NordBord, ForceFrame, DynaMo (VALD Performance, Brisbane, Australia); Perch (Washington, DC, USA); Kineo (Globus, Italy)	Used to assess power output and asymmetries. Used for strength profiling, injury risk screening, and optimizing strength training loads.
Movement assessment & screening	Assess movement patterns, asymmetries, and physical performance indicators	DARI Motion (Kansas City, KS, USA); Brower Timing Systems (Draper, UT, USA); SmartSpeed (VALD Performance, Brisbane, Australia)	Used in profiling and injury risk screenings.
Physiological assessment	Measure hydration, body composition, and biomarkers related to physiological readiness	MX3 Hydration Testing (MX3 Diagnotics, Austin, TX, USA); InBody (Seoul, South Korea); DEXA (dual-energy x-ray absorptiometry), blood diagnostics	Used to monitor hydration status, track body composition, screen for deficiencies, and inform nutrition strategies, training decisions, and competition readiness.
Cognitive assessment	Measure cognitive processing and neurological function	C3 Logix (Cleveland, OH, USA); S2 Cognition (Nashville, TN, USA); Ryzer Test (Urbandale, Iowa, USA)	Used to assess reaction time, decision-making, cognitive function, including baseline concussion assessment for return-to-play.
Video & tactical analysis	Evaluate technical and tactical performance	Volleymetrics (Hudl, Lincoln, NE, USA); Wyscout (Genoa, Italy); HR Tactics (Minneapolis, MN, USA); Exos (Phoenix, AZ, USA)	Used to prepare for games, scout opponents, and deliver player feedback through film sessions.
Continuous physiological monitoring	Track real-time and longitudinal physiological metrics such as sleep, strain/stress, and recovery	WHOOP (Boston, MA, USA); Oura Ring (Oulu, Findland)	Used to support athlete understanding of their own physiology, training responses, and recovery through self-tracking.

Some metrics closely reflect the original data (e.g., peak force), while others combine multiple data points (e.g., player load). As coaching staff discussed these technologies, they often used terms referring to the tools, the data, and the resulting metrics interchangeably. To provide clarity, we distinguish between the tool itself (technology), the values it records (data), and the results it produces (metrics).

Other technology categories arose less frequently. Coaching staff reported using physiological assessment tools, such as hydration and body composition analyzers, to support nutrition and fueling strategies. They also mentioned cognitive assessment tools to evaluate reaction time and decision-making, particularly in the context of concussion return-to-play protocols. Staff also described relying on video and tactical analysis platforms to support opponent scouting, provide player feedback, and guide practice planning through film review.

Coaching staff identified continuous physiological monitoring devices as distinct from other categories due to their athlete-facing nature. These devices, often worn as rings or wristbands, are typically used by student-athletes outside of team settings and collect physiological data independently of team systems. Unlike coach-managed systems, these devices allow student-athletes to self-monitor metrics such as recovery, sleep, and strain (an estimate of physiological load based on recent activity and heart rate response), and choose whether or not to share specific insights with coaching staff. Section 3.3.1 describes how coaching staff navigate the integration of these athlete-managed technologies in practice.

### 3.2 Integration of technology in coaching practices

The following section describes how collegiate coaching staff integrate technology within key coaching domains. These domains refer to common areas of practice in competitive sports that coaching staff referenced when explaining how technology supports their day-to-day work.

**Establishing baselines and benchmarks**—Coaching staff described using technologies to collect foundational data at intake (the initial period when student-athletes first join the team), at the beginning of each season, and at regular intervals to establish baselines and benchmarks (n=4). They commonly use technologies such as force plates and isometric strength testing systems, which generate metrics including jump height, force asymmetry, and hamstring strength. Coaching staff also use markerless movement screening systems that rely on camera-based motion capture to detect asymmetries and identify areas of potential injury risk. These baseline metrics provide a reference point for monitoring progress and tracking changes over time. One strength and conditioning coach [Focus Group 5] explained how intake data informs this process:

“Taking all the info we capture at intake, when they come in as an athlete, and analyze their imbalances, their strengths and weaknesses, their athletic profile, and customizing and trying to enhance those qualities. Our first process is doing a movement analysis using [motion analysis system]-so assessing every bone and joint in the body, then comparing that with normative values, and flagging the top three areas most at risk. From there, we’ll get them on a force plate to generate a force profile. Then we use that information to shape their strength programming.”

**Monitoring athlete performance**—Coaching staff described integrating technology into their daily routines to monitor performance over time and recognize when student-athletes deviate from expected patterns (n=5). They primarily described external metrics such as player load, max sprint velocity, jump mechanics, and hamstring strength. Coaching staff use GPS and IMU systems to capture detailed movement data during both training sessions and competitions. They typically review this information after sessions to assess physical demands and determine whether student-athletes met or exceeded expected thresholds. Staff also mentioned force platforms to regularly measure jump performance and track neuromuscular fatigue. Significant changes in metrics, such as a drop in jump height or a spike in workload, often signal the need to adjust training volume or investigate further. As one sport scientist [Focus Group 2] described:

“If I see big changes in the numbers—like a significant drop in jump height on the force plates—it’s often a sign we need to adjust their workload or check for other issues.”

As part of their broader effort to monitor athlete performance, coaching staff in some focus groups also track hydration status (n=3). They use real-time, non-invasive technologies to assess hydration before practices and competitions, tailoring hydration strategies to individual needs and ensuring student-athletes are physically prepared to perform. A dietitian [Focus Group 3] explained:

“We check hydration levels to make sure they’re ready to go. If they’re not, we treat it with an individualized plan.”

While internal monitoring was less common in our sample, coaching staff across multiple focus groups expressed interest in expanding its use. Some hoped to incorporate more physiological data (n=3), including heart rate and sleep quality, as well as more advanced approaches such as blood biomarker testing. They recognized the potential of internal metrics for monitoring athlete readiness and recovery. However, widespread adoption was hindered by practical challenges including budgetary constraints for advanced testing, like blood work. Additionally, while student-athletes generally comply with team-managed technologies used to track external workload, staff reported lower compliance with continuous wearable devices that collect internal physiological data such as sleep and heart rate variability (n=2). Coaching staff also expressed privacy concerns around continuous or 24-h monitoring (n=3), which further complicates the integration of these technologies in practice. We explore these barriers in greater depth in Section 3.3.

**Individualized student-athlete programming within team environments**—Although all of our focus groups represented team-based sports, where training and practice sessions might be expected to follow a uniform structure, coaching staff emphasized how technology enables individualized adaptations within large team environments (n=5). They use athlete-specific data to tailor training volume, intensity, and recovery strategies. Before a training session, coaching staff review a range of individual-level metrics such as workload trends, jump performance, hydration levels, and recovery status to make targeted adjustments for athletes. These metrics allow coaching staff to determine who is able to push harder, and who may need additional recovery time. By integrating this information into their planning, coaching staff make precise, athlete-specific modifications even within a structured team environment. A strength and conditioning coach [Focus Group 2] explained how they use a percentage-based system to scale workloads dynamically based on student-athlete readiness:

“It’s not uncommon for us to say, ‘Hey, this guy is a 50% guy today, or he’s a 75% guy today,’ based off the prior day’s metrics or some of the testing feedback that we get. So let’s say a player typically plays 30 plays in practice, and he’s a 50% guy—then that coach would script him into 15 reps instead of 30. So we have baseline numbers by position. And then we percentage them out when we are trying to adapt the player load.”

**Return-to-play and injury management**—Coaching staff across all five focus groups described using various technologies to support decisions throughout the rehabilitation process and return-to-play process (n=5). These technologies capture movement and force output, which are used to generate metrics related to postural asymmetries, force deficits, and movement imbalances. Staff rely on these metrics to assess recovery status, adjust training load, and monitor progress. Many noted the importance of having pre-injury baseline data as a reference point for evaluating post-injury status. Coaching staff review athlete data and metrics derived from these technologies at key points in the rehabilitation process to evaluate how student-athletes are responding to interventions and refine their recovery plans accordingly. An athletic trainer [Focus Group 4] described how they monitor jump counts using an IMU unit to guide gradual return to full activity:

“The [IMU tracking system] gives us jump count, which is great for when I’m tracking numbers on jumping for somebody who has an injury. I can look at how many jumps that position typically does, and then adjust their numbers—determine how many they should be doing based on where they’re at in their injury.”

oaching staff also described using cognitive assessment technologies to support return-to-play decisions following concussions. These tools provide data on memory, reaction time, cognitive processing speed, and neurological function, offering quantifiable indicators of recovery. Rather than relying solely on symptom reports, staff use these metrics alongside physical assessments and athlete subjective feedback to inform safer, more confident return-to-play decisions.

**Practice planning**—Coaching staff in our focus groups emphasized the importance of practice planning as a cornerstone of effective coaching, guiding how sessions are structured to ensure student-athletes are prepared to compete at their best. One athletic trainer [Focus Group 5] emphasized that capturing the physical demands of practice sessions is critical to preparing athletes effectively, and described how technology is beginning to support this:

“For over 20 years, how long you practice and how hard you practice is the key. The determining factor to me is what we’re doing in practice physically. I think we can get better information scientifically [through technology] to give us a better number for the load of practice.”

To support this planning, coaching staff use technologies to capture the physical demands of practice sessions and competitions (n=5). GPS and IMU-based tracking technologies collect data on movement patterns, sprint volume, and change-of-direction efforts. As one assistant coach [Focus Group 3] explained:

“I don’t want us to hit change-of-direction every single day. I want us to vary it, right? So I want us to get a certain amount of high-speed accelerations. I want us to get a certain amount of change-of-direction like on certain days. So then, that’s when I build out my training sessions.”

These technologies also use the collected data to calculate workload metrics that reflect both the intensity and duration of an athlete’s activity, providing a single value of how much and how hard the athlete is working. Coaching staff integrate these values into their daily and weekly planning to adjust practice time, manage intensity, and decide which drills or movement types to emphasize.

Beyond daily planning, one focus group discussed using historical workload metrics to prepare their student-athletes for upcoming competitions. Coaching staff described reviewing workload metrics from previous matchups to anticipate the physical demands of similar games. They use these insights to replicate competition-like conditions in practice and gradually build athlete readiness for the demands. One coach [Focus Group 1] explained how this technology-driven approach informed practice planning:

“When you play [x minutes] in a high-intensity game, this is what your typical workload numbers look like. With that in mind, we can say ‘We’ve got a similar game in the three weeks-this is where we need to be at.’”

### 3.3 Goals, motivations, and barriers to technology adoption

At the start of each focus group, we asked the coaching staff about their goals and objectives. Across all five focus groups (n=5), coaching staff consistently emphasized three overarching priorities: keeping athletes healthy, optimizing performance, and supporting holistic student-athlete development. These goals shaped both the motivation to adopt technologies and the practical ways staff integrated them into their coaching routines. As an assistant coach [Focus Group 3] put it:

“To help them [student-athletes] develop incrementally towards the collective goal of winning championships, by individually developing them on and off the field. We focus on their performance as athletes and their development as people.”

While coaching staff recognized the value of technology in supporting these goals, they also pointed out several challenges that can make adoption difficult. This section presents key factors that motivate technology use and the barriers that make it harder for coaching staff to fully integrate these tools into their practices.

#### 3.3.1 Factors that motivate technology adoption

In exploring why coaching staff adopt and continue to use technology, three recurring patterns stood out across the focus groups. First, coaching staff described technologies as tools that enhanced their existing expertise and decision-making. Second, they valued the ability to access data and metrics that were previously unavailable or difficult to quantify. And third, they emphasized that, when used intentionally, technology can strengthen the coach-athlete relationship. The subsections that follow describe each of these motivations in more detail.

**Validating expertise and enhancing decision-making**—Coaching staff adopt technology not to replace their expertise, but to enhance it. In our analysis, references to coaching experience (n=3), coach’s discretion or observation (n=3), and sport-specific knowledge (n=3) all shaped how staff described interpreting and applying outputs of technology. Rather than relying solely on metrics, staff consistently frame technology as a tool that helps them confirm, refine, or challenge what they already observed in their student-athletes. As a coach [Focus Group 5] put it when discussing a biomechanical movement assessment tool:

“I don’t think you ever replace what you see every day, and so those things are, I would say, the most important part, and [motion analysis system] is just supporting that.”

Staff emphasized that their own visual assessments and intuitive judgments remained primary, with technology serving as one of the multiple inputs in the decision-making process. As a coach [Focus Group 4] shared:

“We have to trust our expertise, what we see with our own eyes, and then whatever information we’re getting [from technology] to make the best decisions we can.”

They described using technology to validate what they observed during training and competition, which strengthened their confidence in practice planning and performance expectations. In some cases, unexpected metrics prompted further investigation or changes in workload and recovery plans. In return-to-play contexts, staff valued the ability of technology to provide quantifiable data to complement their clinical assessments, offering clear benchmarks that validate progress and strengthen return-to-play decisions. An athletic trainer [Focus Group 4] explained:

“We give the student-athletes a quantitative number, ‘this is where you were before you were hurt, and this is where we have to get you back to.’”

**Measuring what was previously unavailable and deeper insights**—Coaching staff adopt technology because it provides insights into the physiology, performance, and recovery of student-athletes that was previously unavailable or difficult to measure (n=4). These advancements in tools allow for real-time monitoring of multiple physiological systems without disrupting training or competition, offering deeper and more comprehensive knowledge of how an athlete’s body adapts and responds to training.

An athletic trainer described how motion capture systems and biomechanical tools reveal precise movement inefficiencies and muscular imbalances, helping them refine techniques and reduce injuries. A strength and conditioning coach highlighted how they use force plates to capture detailed force-time data during jump tests, which allow them to analyze stretch-shortening cycles, landing mechanics, and take-off characteristics. These insights directly inform individualized programming in the weight room.

“We use force plates for jump profiles and stretch-shortening cycles, including breaking mechanics and the transition from force production to leaving the ground. Then we reverse engineer strength exercises to enhance those phases, deciding if the athletes need to focus on general strength or more specific strength.”

Some wearable technologies also play a role in capturing insights into sleep performance, heart rate variability, and fatigue, giving coaching staff a clear picture of athlete readiness and recovery needs.

Additionally, body composition assessments allow for precise measurements of muscle mass distribution, fat percentage, and bone density, offering clearer measures for nutrition programming. As one dietitian [Focus Group 5] noted, these metrics allowed nutrition conversations to move beyond subjective impressions:

“Nutrition is aesthetic-based, you know, some folks say ‘oh, I feel like I’m getting bigger’ but it’s good to see the numbers-wise as well.”

By adopting these technologies, coaching staff access new forms of data that support deeper insights into student-athlete health, performance, and development.

**Enhancing the coach-athlete relationship through technology**—Coaching staff across focus groups described adopting technology not only to support performance optimization but also to strengthen the coach-athlete relationship. They explained that when used intentionally, technology offers new ways to structure conversations, provide transparent feedback, and reinforce shared goals between coaching staff and student-athletes.

Coaching staff in three focus groups emphasized that objective performance metrics help guide discussions with student-athletes, offering a clarifying way to explain training decisions or why adjustments are needed (n=3). A strength and conditioning coach [Focus Group 4] described how sharing data supports athlete buy-in:

“For the athletes I usually sit down with them and go through the information, ‘Here’s why we are working on this.’”

Coaching staff also noted that displaying metrics such as speed, jump height, or who covered the most distance in practice helps motivate athletes to push themselves during drills or testing sessions (n=5). For some teams, staff distribute wearable devices in the form of rings and wristbands that allow student-athletes to independently monitor their own physiological data, such as sleep, heart rate variability, and strain. This allows them to reflect on how training affects their recovery and overall physical state. One assistant coach [Focus Group 3] described how these technologies sometimes open up a check-in:

“If I know they had a really high load on [GPS/IMU system] or just from the ‘eye test’ they look like they’re dragging, I’ll ask them about their recovery score [derived from the athlete’s continuous wearable device] and how they’re feeling.”

Other staff described how technology can serve as a relational bridge, helping them spot subtle declines in metrics that might signal underlying physical or emotional strain. These signs often prompt individual follow-ups or referrals to additional resources (n=3). A strength and conditioning coach [Focus Group 2] reflected:

“If we see someone’s numbers are down, we can ask them if something’s going on and uncover things. It allows us to send them to the training room or make sure they’re getting extra treatment.”

Even as they value how these data-driven elements add to the coach-athlete relationship, coaching staff consistently emphasized that technology does not replace personal relationships (n=4). As one coach [Focus Group 5] summarized:

“It’s always about the people and your connection with the athlete. Can you get them to do what you want them to do? Can they see and buy into what you’re trying to incorporate with the data?”

Across roles and focus groups, coaching staff described these relational benefits as a compelling reason to adopt technology in their programs.

#### 3.3.2 Barriers to effective adoption

Although coaching staff see value in technology within collegiate sports, they also described several challenges that make it difficult to adopt or use effectively. These barriers were not framed as resistance to innovation, but as reflections of the practical and structural realities of integrating technology in environments where funding varies by team, technologies often lack sport-specific adaptability, support staff are limited, and student-athlete well-being remains a core priority. The subsections that follow detail these barriers as described across the focus groups.

**Financial constraints, accessibility, and departmental limitations**—One of the most immediate barriers to adoption described by coaching staff was the financial investment required to acquire and maintain advanced sports technology (n=2). Many of the technologies mentioned such as GPS/IMU tracking systems, force plates, and motion-capture technologies are expensive, and funding levels vary across teams and programs. Even in focus groups that included staff from different sports teams within the same university, it was evident that well-established and well-resourced programs tend to use a greater number and variety of technologies. One head coach explained the reality of budget constraints and limited equipment availability, noting there were *“not enough sensors for the entire team,”* which often forced staff to prioritize which athletes received access (n=2). This was also extended to more advanced testing such as blood biomarker analysis, which was only available to select athletes due to cost. A coach [Focus Group 4] described switching from one technology to another because of financial and departmental constraints:

“The reason we started using it is because our department made that decision. It wasn’t a team-level choice. This was a departmental decision. For us, it’s largely about budget. We don’t have the kind of budget that other sports might have to try out the newest technology just to see how it goes. The department said this is the technology we’re going to use, so that’s what we use. Are we married to it? No. It’s just the one we can afford because it doesn’t cost us anything.”

This resulted in a loss in longitudinal data, as historical performance trends often can not be easily transferred between or interpreted across technologies.

**Mismatch between technology and sport-specific needs**—While technology generates valuable insights, coaching staff noted that many tools cannot adapt to the specific demands of their sport, limiting their effectiveness (n=3). Many of the technologies used across multiple teams often lacked built-in customizations for different positions, movement patterns, and practice environments. As a result, coaching staff frequently have to interpret or adjust the metrics themselves to ensure their relevance. For example, a volleyball coach described how workload metrics need to be filtered through understanding positional differences for effective use since hitters perform more jumps than other players:

“We look at it by position. Our hitters did this, our middles did this.”

Similarly, a member of the basketball coaching staff emphasized the same notion that positional demands need to be considered to extract meaningful insights:

“A big is not going to be running as much as a guard.”

Coaching staff also expressed uncertainty about how well current technologies capture the actual demands placed on student-athletes (n=2). Even when athletes completed the same drills in the same practice, staff observed unexpected variability in workload metrics. This raised questions about whether the tools accounted for subtle differences in athlete movements or whether the data were consistently reliable. An athletic trainer [Focus Group 1] said:

“We will have one or two, where they’ll run through the exact same practice but their numbers will be sky high and the rest of the crew will be average, you know, and why is that?”

Similarly, GPS and IMU tracking systems that monitor external load may not capture the full movement demands of positions, such as a soccer goalie or volleyball players who dive or rely on upper body force production since these metrics are primarily influenced by locomotive activity.

Environmental conditions present another challenge. A coach described how soft or uneven fields increase the physical demands on athletes, but GPS-based workload metrics, driven by movement data, do not (to their knowledge) reflect this added effort. Because the metrics rely on values such as acceleration and displacement, they may underestimate workload when athletes move more slowly due to surface resistance, despite exerting more internal effort or muscular force. To their knowledge, the technology offers no built-in way to adjust for terrain differences. In these cases, coaching staff rely on their own judgment to interpret the data in context or adjust their understanding of the metrics to better align with the physical effort they observe during training or competitions.

**Growing need for interdisciplinary experts and support staff**—As technology becomes more advanced and embedded in collegiate athletics, its effective adoption increasingly depends on staff who can manage, interpret, and integrate data into coaching workflows. Coaching staff in our focus groups noted that without dedicated personnel to handle these tasks, these responsibilities fall on them, limiting their ability to use the tools effectively and focus on coaching their student-athletes (n=4).

Coaching staff in three focus groups mentioned interns as essential to the daily operations of technology within their teams (n=3), although these staff are often temporary and less experienced. Interns assisted with setting up equipment, capturing practice data, and generating daily and weekly reports, ensuring that information was readily available to inform decisions.

Beyond interns, more advanced roles have emerged to optimize the use of data and technology in collegiate athletics. Coaching staff frequently mentioned the Director of Sports Performance and Analytics as a pivotal figure who coordinates testing and assessments, oversees interns, and analyzes trends from systems like motion capture or force plate technologies. One athletic trainer [Focus Group 5] explained their reliance on this role:

“There are a lot of things I don’t understand how to do, and then to be completely honest, it’s not an excuse, I don’t even want to learn. But I trust him immensely with the information that he’s given me.”

Other roles, such as tech coordinators and directors of video analysis, further illustrate the growing infrastructure that supports the journey from data collection to decision-making. Staff described tech coordinators who log and tag practice data, and a director of video analysis who creates customized film cuts to assist in evaluations and tactical planning (n=3). Coaching staff also described collaborations with professors and academic researchers as additional support, who contribute research-driven perspectives to practical applications in coaching (n=3). Together, these roles and partnerships illustrate the expanding network of personnel required to integrate technology effectively in collegiate sports environments.

**Potential burden on student-athletes**—Coaching staff in our focus groups reflected on ways that technology, if not used thoughtfully, could introduce unintended consequences for student-athletes (n=5). They expressed concerns that some uses of technology can introduce stress or *“get in their head,”* shifting the student-athlete’s focus away from the game itself (n=4). Others were concerned that over-analysis and fixation on metrics may cause athletes to overreact if information does not meet expectations (n=3). Additionally, one coach noted excessive or repetitive testing as a barrier. When testing becomes too frequent or lacks clear value to student-athletes, coaches observe that engagement often diminishes, leading to a reduction in effort during assessments. This, in turn, affects data quality and limits the insights that can be drawn from it. One strength and conditioning coach [Focus Group 5] reiterated:

“It is only as accurate as the athlete, the effort has to be there.”

Another staff member [Focus Group 5] added:

“Time is a big resource and if we’re spending so much time doing tests that aren’t giving value to the athletes, I think we kind of miss the connection there. We want this to be purposeful for them so that they’re seeing value in this. Not asking ‘I gotta do another test?’”

Coaching staff also raised concerns about the use of continuous physiological monitoring devices, particularly those worn outside of practice (n=2). Several staff members reported intentionally avoiding full access to information from these types of technologies to prevent unnecessary intrusion into student-athletes’ daily lives. While they acknowledged that the metrics could offer valuable insights, they noted that constant surveillance, even with good intentions, could shift technology from a tool of support to a source of pressure.

In addition to concerns about monitoring, staff also discussed how information from technology is shared with student-athletes. When asked whether student-athletes have access to their own metrics or reports, specifically from those generated by coach-managed technologies, several coaches expressed hesitation. They worried that without proper context, even accurate metrics could be misinterpreted and lead to unnecessary stress or unproductive reactions. An assistant coach [Focus Group 3] explained:

“Any statistic can tell a story, if [athletes] see they don’t hit it for one game or training, they might go off the wall.”

To manage this, some staff noted that they filter or simplify the information they share (n=4), aiming to keep the insights useful and motivating without overwhelming the athlete.

## 4 Discussion

This study explored how collegiate coaching staff utilize various technologies within their coaching practices. Our findings demonstrate that modern coaching incorporates diverse technological tools and has progressed significantly. These technologies are being applied more extensively and across a broader set of coaching domains than what has been described in existing literature, particularly within collegiate sports settings. The proliferation of GPS/IMU-based trackers, force plates, and physiological monitoring devices has enabled coaching staff to gain nuanced insights into athlete performance, facilitating more precise and informed decisions in training and athlete management.

A notable insight from our research is that while technology provides valuable quantitative insights, it fundamentally complements rather than replaces core coaching expertise. Coaching staff consistently emphasized that their visual assessment, intuition, and sport-specific knowledge remain central to effective coaching. They integrate technology primarily to validate, refine, or challenge existing coaching observations. This reflects a meaningful shift from earlier studies, such as Liebermann et al. (13), in which fewer than two-thirds of experienced coaches reported using technology in coaching. Notably, Liebermann’s participants ranked “having a good relationship with athletes” and “observing relative improvements in performance” as higher priorities over data-driven measures. Our findings extend this perspective in a contemporary context, illustrating that even as technology becomes more integrated into coaching environments, the values that guide its use remain deeply rooted in human connection and coaching expertise.

At the same time, technological competence is becoming a new core skill for modern coaching. According to the International Council for Coaching Excellence (ICCE) framework (22), effective coaching encompasses three key areas: professional knowledge (e.g., understanding sport-specific demands, managing athlete development), interpersonal knowledge (e.g., supporting communication and relationship-building with the athlete), and intrapersonal knowledge (e.g., promoting reflective practice and ongoing learning). Our findings suggest that working with technology now spans across all three, as described in the following paragraphs.

In area of professional knowledge, a significant finding is the role technology plays in supporting highly personalized athlete management within structured team environments. Coaching staff described dynamically adjusting training or recovery strategies based on real-time or recent data specific to each student-athlete. Despite managing teams with many student-athletes, this approach, supported by baseline metrics and daily monitoring, underscores technology’s potential to provide greater personalization in training and care, helping staff tailor support to individual needs within large team settings.

Furthermore, within the interpersonal knowledge area, our findings reveal that technology is now embedded in the relational aspects of coaching, and that communication about data is an emerging core competency area in coaching. Coaching staff utilize technology-driven insights as opportunities for structured conversations with student-athletes, offering clarity, motivation, and reassurance, particularly during injury recovery or high training demands. However, they also recognize the potential psychological burdens that poorly contextualized data could impose on athletes. Coaching staff carefully select which metrics to share, aiming to maintain motivation and focus without causing unnecessary stress or cognitive overload. This aligns with recent studies advocating a balanced approach to data transparency and athlete autonomy (23, 24) and with prior research highlighting the role of coaches in managing sensitive athlete data and mitigating emotional burden (6).

Moreover, in the area of intrapersonal knowledge, coaching staff must now engage in greater self-reflection about how to meaningfully use technology and how it fits into their ongoing learning as coaches. Our findings highlight that sport-specific adaptability of technologies presents a barrier to effective adoption. Coaching staff described the need to interpret and tailor metrics to the distinct demands of different sports, positions, and training environments. This challenge underscores the growing importance of data literacy, which involves developing the ability to work with data in context, including interpreting metrics, spotting patterns, recognizing misleading trends, and effectively communicating the information. These are core competencies described in data literacy frameworks (25) that are only beginning to be applied to sports contexts (23), emphasizing the need for more structured approaches to data education in coaching. While some staff in our study embrace these skills, others rely on trusted personnel to provide key insights, revealing a spectrum of data fluency. Integrating data literacy into coach education and sports science curricula will be critical to ensuring the next generation of coaches can confidently engage with technology and apply it meaningfully.

As sports technology becomes more advanced and integrated across all aspects of coaching, it is impractical for coaching staff to manage it alone. This has created a need for dedicated personnel who assist with tasks of data collection, processing, and reporting. Our findings illustrate how roles such as interns, tech coordinators, and performance analysts are becoming essential within high-performance environments, helping offload the technical burden from coaches and ensuring timely, actionable insights. Interdisciplinary collaboration is already a foundational aspect of high-performance sport, with distinct but interdependent roles working together to support athlete development (26). We saw this reflected in the composition of our focus groups, which included sports coaches, athletic trainers, strength and conditioning staff, dietitians, and other professionals. However, our findings indicate that this interdisciplinary team is continuing to expand in response to the increasing role of technology. Coaching staff described the addition of new roles, such as the Director of Performance and Analytics, as well as collaborations with research scientists. We suggest that professionals with expertise in both computer science and sports science are uniquely positioned to bridge the gap between technology and coaching. These hybrid experts can play a key role in ensuring technology is both accessible and useful for coaching staff in high-performance environments.

Finally, as technology becomes more embedded in daily coaching routines, our findings suggest that ethical and institutional considerations must also evolve. Coaching staff expressed concerns about the psychological impact of over-monitoring and questioned how frequently testing should occur, what data were necessary, and how those data were communicated to student-athletes. While many coaches exercised discretion in data sharing, our findings did not reveal any mention of formal guidance on how to navigate these decisions. These observations point to a need for broader institutional support such as ethics committees, coach training on data privacy, and clear policies around technology use that can help guide both athlete protection and coaching practice.

## 5 Limitations and future work

While this study provides extensive insights into the landscape of sports technology in collegiate sports, several limitations should be acknowledged.

First, our findings reflect only the coaching staff’s perspectives and do not include the viewpoints of the student-athletes or other key stakeholders, such as those involved in the technology management (interns, director of performance analytics), administrators, and sports technology developers. Additionally, this study focuses on a single, well-resourced Division I university in the United States, meaning that the results may not be generalizable to other levels of sport. Future research should expand to diverse coaching environments and varying levels of technological infrastructure to determine whether these findings apply more broadly.

Second, our study included only coaching staff from team-based sports, meaning that sports with different performance structures, such as individual time-based sports, like track and field or swimming and diving, may engage with technology in fundamentally different ways. Technologies such as GPS/IMU-based trackers, which were mentioned often in our study, may be less relevant in these contexts, where alternative metrics may be prioritized. Future research should explore how coaching staff in these sports adopt, adapt to, and/or resist technology.

Finally, while discussions naturally revealed perspectives on data literacy and adoption challenges, we did not explicitly ask coaches to reflect on their own training gaps or ideal educational resources for navigating technology in sports. Future research could address this by directly gathering coaching staff perspectives on their preparedness and identifying areas where formal training or institutional support could enhance their technology integration skills.

## 6 Conclusion

This study provides an in-depth examination of how collegiate coaching staff integrate technology into their coaching practices, contributing to the broader understanding of digital tools in sports coaching. Our findings affirm that technology is increasingly embedded in coaching practices, enhancing decision-making, optimizing athlete management, and supporting coach-athlete communication. These technologies serve as an extension of coaches’ intuition, experience, and sport-specific knowledge.

We have identified both opportunities and challenges in technology adoption. Technology provides opportunities for deeper insights into performance and injury prevention, enabling coaches to measure what was previously unavailable and enhance precision in decision-making. At the same time, effective use of technology in coaching requires thoughtful integration of data, careful communication of metrics, and sensitivity to the relational dynamics involved in applying the insights with student-athletes. Coaching staff must now strategically integrate technological insights in ways that inform and motivate athletes while mitigating risks of cognitive overload or misinterpretation.

These findings suggest that, moving forward, as coaching staff integrate technology across a growing range of domains, coaching will require targeted strategies to improve data literacy, expand interdisciplinary support through technology specialists, and establish ethical guidelines for technology use in sporting environments. The insights presented here may have broader implications beyond collegiate sports, offering valuable direction for scholars, coaches, and organizations aiming to strengthen coaching practice and athlete outcomes through thoughtful integration of technology.

## Data Availability

The data analyzed in this study is subject to the following licenses/restrictions: The dataset consists of qualitative transcripts from human subject research and is not publicly available due to confidentiality agreements and institutional ethics protocols. Participants did not consent to public data sharing, and data includes potentially identifiable information. Requests to access these datasets should be directed to mollie.brewer@ufl.edu.
